# Sinoacutine inhibits inflammatory responses to attenuates acute lung injury by regulating NF-κB and JNK signaling pathways

**DOI:** 10.1186/s12906-021-03458-0

**Published:** 2021-11-20

**Authors:** Yuancui Zhao, Lili Cui, Xing Xin Yang, Xingqian Sun, Yunkuan Liu, Zixian Yang, Liyuan Zhu, Chaorui Peng, Danye Li, Junfei Cai, Yunshu Ma

**Affiliations:** 1 School of Chinese Material Medicine, Yunnan University of Chinese Medicine, Kunming, 650500 China; 2 Yunnan Key Laboratory of Dai and Yi Medicine, School of Chinese Material Medicine Yunnan University of Chinese Medicine, Kunming, 650500 China; 3 Key Laboratory of External Drug Delivery System and Preparation Technology in University of Yunnan, Kunming, 650500 China; 4 School of Pharmacy, Nanjing University of Chinese Medicine, Nanjing, 210046 China; 5 Key Laboratory of Southern Medicine Utilization, Kunming, 650500 China

**Keywords:** Sinoacutine, Anti-inflammatory, RAW264.7 macrophages, NF-κB, MAPK, Acute lung injury

## Abstract

**Background:**

*Stephania yunnanensis* H. S. Lo is widely used as an antipyretic, analgesic and anti-inflammatory herbal medicine in SouthWest China. In this study, we investigated the anti-inflammatory activity and mechanism of sinoacutine (sino), one of the primary components extracted from this plant.

**Methods:**

A RAW264.7 cell model was established using lipopolysaccharide (LPS) induced for estimation of cytokines in vitro, qPCR was used to estimate gene expression, western blot analysis was used to estimate protein level and investigate the regulation of NF- κB, JNK and MAPK signal pathway. In addition, an acute lung injury model was established to determine lung index and levels of influencing factors.

**Results:**

Using the RAW264.7 model, we found that sino reduced levels of nitric oxide (NO), tumour necrosis factor-α (TNF-α), interleukin (IL)-1β and prostaglandin E_2_ (PGE_2_) but increased levels of IL-6. qPCR analysis revealed that sino (50, 25 μg/ml) inhibited gene expression of nitric oxide synthase (iNOS). western blot analysis showed that sino significantly inhibited protein levels of both iNOS and COX-2. Further signalling pathway analysis validated that sino also inhibited phosphorylation of p65 in the NF-κB and c-Jun NH2 terminal kinase (JNK) signalling pathways but promoted the phosphorylation of extracellular signal regulated kinase (ERK) and p38 in the MAPK signalling pathway. In addition, in a mouse model induced by LPS, we determined that sino reduced the lung index and the levels of myeloperoxidase (MPO), NO, IL-6 and TNF-α in lung tissues and bronchoalveolar lavage fluid (BALF) in acute lung injury (ALI).

**Conclusion:**

Taken together, our results demonstrate that sino is a promising drug to alleviate LPS-induced inflammatory reactions.

**Supplementary Information:**

The online version contains supplementary material available at 10.1186/s12906-021-03458-0.

## Background

Inflammation, the basis of acute and chronic syndromes, often plays a crucial role in acute respiratory distress syndrome (ARDS), atherosclerosis, inflammatory bowel disease, el at [1].. Lipopolysaccharide (LPS) can stimulate macrophages to secrete various inflammatory cytokines, further causing systemic inflammatory reactions that lead to shock and sepsis in severe cases, eventually progressing to multiple organ dysfunction [2]. Drugs currently used for the treatment of inflammation are classified into steroidal anti-inflammatory drugs (SAIDs) and nonsteroidal anti-inflammatory drugs (NSAIDs). However, after many years of clinical use, it was found that long-term use of SAIDs could lead to complications such as adrenal cortex dysfunction [3], while NSAIDs, represented by aspirin, have good anti-inflammatory effects and do not cause some of the adverse reactions induced by SAIDs. However, NSAIDs also trigger a series of other adverse reactions such as damage to the liver and digestive tract after long-term use [4]. Therefore, identifying novel anti-inflammatory drugs with high efficacy and minimal side effects has become a hot spot in the research and development of lead compounds [5] .

Acute lung injury (ALI) and the more severe acute respiratory distress syndrome (ARDS), a subtype of ALI characterized by more severe hypoxemia, are the pulmonary manifestations of an acute systemic inflammatory process clinically characterized by pulmonary infiltrates, hypoxemia and oedema [6]. A prospective, population-based, cohort study in 21 hospitals demonstrated that the in-hospital mortality rate of ALI was 38.5% [7]. Studies have demonstrated that the development of ALI/ARDS leads to excessive production of proinflammatory cytokines, such as tumour necrosis factor (TNF)-n, interleukin (IL)-1rl IL-6, and IL-8, by immune cells [8], and chemotactic inflammatory cells excessively infiltrate lung tissue, resulting in oedema and gas exchange deterioration [9]. These phenomena indicate that acute inflammation plays an important role in the ALI/ARDS process16. In recent decades, mechanical ventilation has been conventionally used as a standard treatment method, but there is still no feasible or effective treatment method to treat ALI that reduces the mortality rate in critically ill patients [10]. Therefore, treatment of the primary disease and control of the systemic inflammatory response has become a major therapeutic strategy. Some natural ingredient have been reported to exert protective effects on LPS-induced ALI in mice and in RAW264.7 cells and have potential as therapies for the treatment of pulmonary inflammation. Such as Plantamajoside [11], Dehydrocostus lactone and Astaxanthin. Hence, developing more effective strategies to inhibit inflammatory responses and identifying new diagnostic and therapeutic targets are critical for improving patient outcomes [12, 13].

Stephania yunnanensis H. S. Lo is a plant of Subgen. Tuberiphania Lo et M. Yang, Genus Stephania from Yunnan province, China. Many species of plants in Subgen. Tuberiphania are traditional Chinese herbal medicine and ethnic medicines with a long history of medical application and are widely distributed throughout South and West China, especially in Yunnan, Guizhou and Sichuan provinces. They have served as ethnic drugs in Yi (Biwugao), Dai (Bobohan) and other nationalities in Southwest Chinese populations with antipyretic, analgesic and anti-inflammatory actions [14–16]. Sinoacutine (sino) is a morphinoid alkaloid extracted from *S. yunnanensis* S. H. Lo [17] and *S. epigaea* H.S.Lo [14]. It has been reported that sino reduces articular swelling in an anti-rheumatoid arthritis model caused by type II collagen in rats [18], validating that sino exert an anti-inflammatory effect in vivo. Another study [19] found that sino plays an analgesic role by increasing the hot plate-induced pain threshold in mice, reducing the twisting times of mice caused by acetic acid, and increasing the pain threshold in response to electrical stimulation in the mice toes. At the same time, it also coordinates and enhances the sedative and hypnotic effects of sodium pentobarbital [19]. The structure of sino is similar to that of sinomenine (sin), a marketed medication in China used for the treatment of osteoarthritis and rheumatoid arthritis. It was reported that sin has strong anti-inflammatory and immunosuppressive effects, as well as analgesic and sedative effects [20], through inhibiting the NF-κB [21] and MAPK [22] signal transduction pathways. In addition, Liu et al. [14] found that sin attenuates ALI by suppressing inflammation. Therefore, it has been speculated that sino has similar effects as sin with respect to inhibiting the inflammatory signalling pathway and the expression of inflammation factors, and it is necessary to compare the difference in activity and mechanism between these two compounds toidentify new anti-inflammatory lead components with high efficiency and minimal side effects.

In this study, we first explored the anti-inflammatory effect and potential mechanism of sino in vitro using RAW264.7 mouse macrophages stimulated with LPS. Then, an ALI mouse model induced by LPS was established to investigate the anti-inflammatory effect in vivo to provide a theoretical basis for further research and novel drug development.

## Methods

### Drugs and reagents

The sino (96.46%) used in this experiment was prepared by our laboratory. Dexamethasone sodium phosphate injection (Dex) was purchased from Tianjin Suicheng Pharmaceutical Co., Ltd. (Tianjin, China). Zhengqingfengtongning Injection(sin) was produced by Zhengqing Pharmaceutical Co., Ltd. (Hunan, China). The NO Assay Kit (nitrate reductase method) and β-actin antibody were purchased from Nanjing Jiancheng Bioengineering Institute (Jiangsu Sheng, China). High glucose Dulbecco’s Modified Eagle’s Medium (DMEM), fetal bovine serum (FBS), and phosphate-buffered solution (PBS) were obtained from Biological Industries (Kibbutz Beit Haemek, Israel). Cell Counting Kit-8 (CCK-8) was purchased from TransGen Biotech (Beijing, China). Dimethyl sulfoxide and LPS were purchased from Sigma (St. Louis, MO, USA). The antibodies specific for iNOS, phosphorylated p65 (p-p65), p65, p-IκB, p-ERK, ERK, P-P38, P38, P-JNK, JNK and horseradish peroxidase-conjugated goat anti-rabbit secondary antibody were purchased from Cell Signaling Technology (Danvers, MA, USA). The enhanced BCA Protein Assay Kit was purchased from Beyotime Biotechnology (Beijing, China). The mice TNF-α, IL-6, and myeloperoxidase (MPO) enzyme-linked immunosorbent assay (ELISA) kits were purchased from multi sciences (Hangzhou, China). The Total RNA Extraction Kit, GoScript™ Reverse Transcription System, and GoTaq® qPCR Master Mix were purchased from Shanghai Promega (Shanghai, China).

### Plant material

The plants of *Stephania yunnanensis*. Lo were collected from Yun county, Yunnan Province, China, and identified by Professor Yunshu Ma (College of Pharmaceutical Science, Yunnan University of Chinese Medicine) [23], which is not an endangered species, hence no special governmental permission was required for collection. The specimen was deposited in the College of Pharmaceutical Sciences, Yunnan University of Chinese Medicine. The root tuber of *Stephania yunnanensis*. Lo was used to isolated sino.

### Extraction and separation of sino [24]

Collection of *Stephania yunnanensis*. Lo was sun-dried and pulverized into coarse powder, refluxed and extracted in 95% ethanol 3 times to obtain the extract. Extraction was performed with acid-soluble chloroform, and the PH value was adjusted to 8–9 with alkali solutions. The extract was dissolved and mixed with a silica gel column (petroleum ether-acetone system gradient elution, chloroform-methanol system gradient elution, repeated elution), and an LH-20 dextran gel column (chloroform-methanol 1:1) was separated and purified several times to obtain colourless crystals. The structure was identified using mass spectrometry, proton nuclear magnetic resonance (^1^H NMR), and carbon-13 NMR (^13^C NMR) analyses. Content determination was performed by HPLC.

### Cell culture

RAW264.7 cells were purchased from the Cell Resource Center of Shanghai Institute of Life Science, Chinese Academy of Sciences (Shanghai, China). RAW264.7 cells were cultured in high glucose DMEM supplemented with 10% FBS and 1% penicillin-streptomycin in a humidified incubator at 37 °C and 5% CO2.

### CCK-8 assay

RAW264.7 cells (2.5 × 10^5^ cells/ml) were seeded in 96-well plates and cultured for 24 h in the presence of 5% CO2 at 37 °C. Sin (50 mg/2 ml) was diluted to 100 mg/ml in DEME medium. Since sino is difficult to dissolve in DMEM medium, 8 μg/ml DMSO was first used to dissolve it, and then DMEM medium was used to dilute it to the corresponding concentration. Then, the cells were treated with 100 μl sino at different concentrations (25, 50, 100 μg/ml) and DMSO (8 μg/ml) for 24 h, and 10 μl CCK-8 was added to each well and the cells were cultured for 3 h, the OD at 450 nm was measured by a microplate reader. and the cell viability and concentrations for experiment was calculated.

### NO and inflammatory cytokine measurement

RAW264.7 cells (2.5 × 10^5^ cells/well) were seeded into 24-well plates. Then, cells were treated with sin, Dex, and different concentration of sino for 2 h followed by incubation with 1 μg/ml LPS for 24 h. The concentrations of cytokines NO, TNF-α, IL-1β, IL-6 and PGE_2_ in the supernatant were detected according to the manufacturer’s instructions. All experiments were repeated for at least three times.

### Quantitative real-time PCR

RAW264.7 cells (8 × 10^5^ cells/well) were plated into 6-well plates. Then, the cells were treated with sin, Dex, and different concentrations of sino for 2 h followed by incubation with 1 μg/ml LPS for 6 h. Total RNA extraction and isolation was performed, and the mRNA levels were measured using quantitative real-time PCR (qPCR). Relative gene expression was normalized to GAPDH. Pre-denaturing was performed at 95 °C for 10 min followed by 40 cycles under conditions of denaturation at 95 °C for 15 s and annealing at 60 °C for 1 min. All experiments were repeated for at least three times. Primers are listed in Table 1.

**Table 1:** Primer sequences used in this study

Gene	Size(bp)		Sequences (5′-3′)
iNOS	109	Forward	TGCCACGGACGAGACGGATAG
		Reverse	CTCTTCAAGCACCTCCAGGAA
COX-2	81	Forward	GGTGCCTGGTCTGATGATGTATGC
		Reverse	GGATGCTCCTGCTTGAGTATGTCG
GAPDH	183	Forward	GGTTGTCTCCTGCGACTTCA
		Reverse	TGGTCCAGGGTTTCTTACTCC

### Western blot analysis

RAW264.7 cells (8 × 10^5^ cells/well) were plated into 6-well plates. Then,the cells were treated with sin, Dex, and different concentrations of sino for 2 h followed by incubation with 1 μg/ml LPS for 24 h. A cell lysis buffer (PMSF:RIPA = 1:100) was used to extract total proteins, and the protein concentrations were determined using a BCA protein assay kit. The protein extracts (40 μg) were separated using 10% SDS–PAGE and electrotransferred to PVDF membranes, which were incubated with primary antibody according to the conditions shown in Table 2. After incubation, the membranes were washed in TBST, and then incubated with horseradish peroxidase-conjugated goat anti-rabbit secondary antibody. The signals were detected using an enhanced chemiluminescence kit.

**Table 2:** PVDF membrane antibody incubation conditions

	iNOS, p65, p-IκB, ERK, p-JNK, JNK, β-Tublin, β-actin, GAPDH, COX-2	p-p65, p-ERK, p-p38
Blocking time	1 h	12 h
Antibody incubation time	12 h	1 h

### Animal experiment

Kunming (KM) mice (male, 20 ± 2 g) were purchased from Hunan Skolek Jingda Experimental Animal Co., Ltd. (Reg. No. SCXK (Hunan) 2016–0002) and fed a normal diet. All animal protocols were approved by the Yunnan University TCM Committee on Animal Care and Use (No. R-062016002).

One hundred and four mice were randomly divided into seven groups: control (saline), model (LPS, 10 mg/kg), sin (13 mg/kg), Dex (7 mg/kg), and 3 doses of sino (3, 6, and 12 mg/kg). LPS was injected into the tail vein except in the control group. After 12 h, the control and model groups were injected with the same dose of saline, while the 5 treatment groups were injected with their corresponding drugs. After 12 h, the trachea of six mice in each group was exposed, and their left lungs were lavaged with saline two times to obtain BALF sample [25], mice were inhaling isoflurane (2%) to anesthetize before sacrifice. The lung tissues of the remaining mice were collected, weighed and homogenized.

### Measurement of lung index, NO, MPO, IL-6, TNF-α levels

The NO, MPO, IL-6, TNF-α contents in BALF and/or lung tissues were tested using kits according to the manufacturer’s protocols. After the lungs were weighed, the lung index (LI) was calculated according to the following formula to evaluate the degree of pulmonary edema.

$$\mathrm{LI}=\left(\mathrm{lung}\ \mathrm{weight}\right)/\mathrm{weight}\times 100\%$$

### Histopathological evaluation of the lungs

The right lungs of mice in each group were collected and fixed in 4% paraformaldehyde solution, embedded in paraffin, cut into 3 μm sections and stained with haematoxylin/eosin (H&E). Finally, the pathological changes in the tissues were microscopically examined.

### Statistical analysis

SPSS19.0 was used to analyze the differences between values. All values were expressed as means ± SDs. The difference between two groups was determined to be statistically significant using one-way ANOVA (Dunnett’s *t* test). Statistical significance was accepted at *p* < 0.05 or *p* < 0.01.

## Results

### ^1^H NMR and ^13^C NMR analyses

**Fig. 1:**
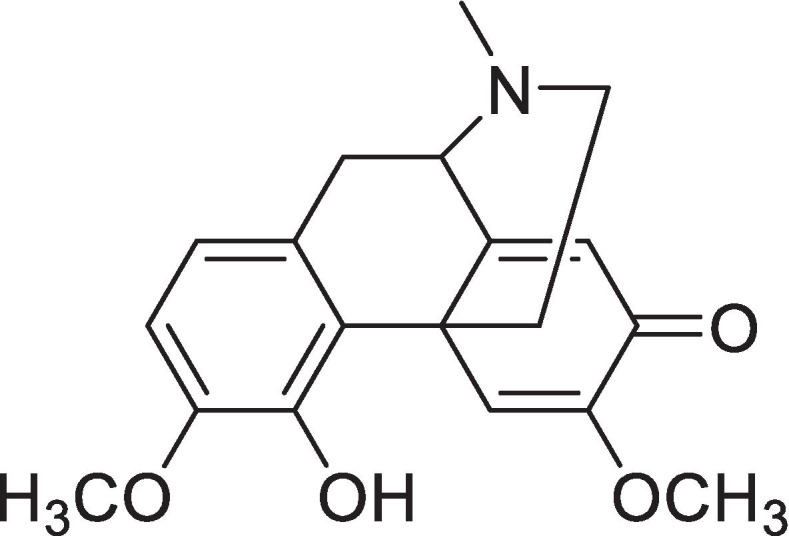
The chemical structure

The substance is colorless massive crystal (methanol). mp195–198 °C, MSm/z327 (M^+^); C_19_H_21_O_4_N. ^1^H-NMR (CDCl_3_, 400 MHz): δ_H_: 1.77 (1H, td, H_a_-15), 2.37 (1H, *J* = 2.70 Hz, H_b_-15), 2.51 (1H, d, *J* = 2.76 Hz, H_a_-16), 2.64 (1H, dd, *J* = 3.40 Hz, *J* = 3.01 Hz, H_b_-16), 2.46 (3H, s, N-CH_3_), 3.00 (1H, dd, *J* = 5.48 Hz, *J* = 5.50 Hz, H_a_-10), 3.36 (1H, d, *J* = 17.71 Hz, H_b_-10), 3.46 (1H, dd, *J* = 5.48 Hz, *J* = 5.50 Hz, H_a_-10), 3.36 (1H, d, *J* = 17.71 Hz, H_b_-10), 3.46 (1H, s, H-9), 3.74 (3H, s, 3-OCH_3_), 3.87 (3H, s, 6-OCH_3_), 6.31 (1H, s, 4-OH), 6.74 (1H, d, *J* = 8.31 Hz, H-1), 6.65 (1H, d, *J* = 8.28 Hz, H-2), 6.32 (1H, s, H-5), 7.53 (1H, s, H-8); ^13^C-NMR (CDCl_3_, 400 MHz): δ_c_: 120.3 (d, C-1), 109.5 (d, C-2), 145.4 (s, C-3), 143.3 (s, C-4), 118.9 (d, C-5), 160.8 (s, C-6), 181.3 (s, C-7), 122.5 (d, C-8), 61.0 (d, C-9), 37.3 (t, C-10), 129.3 (s, C-11), 123.7 (s, C-12), 43.5 (s, C-13), 150.9 (s, C-14), 32.6 (t, C-15), 47.0 (t, C-16), 41.6 (q, N-CH_3_), 56.3 (q, 3-OCH_3_), 54.8 (q, 6-OCH_3_). the purity was 96.46% by HPLC. The chemical structure was listed in Fig. 1.

### Effects of sino on cell viability

**Fig. 2:**
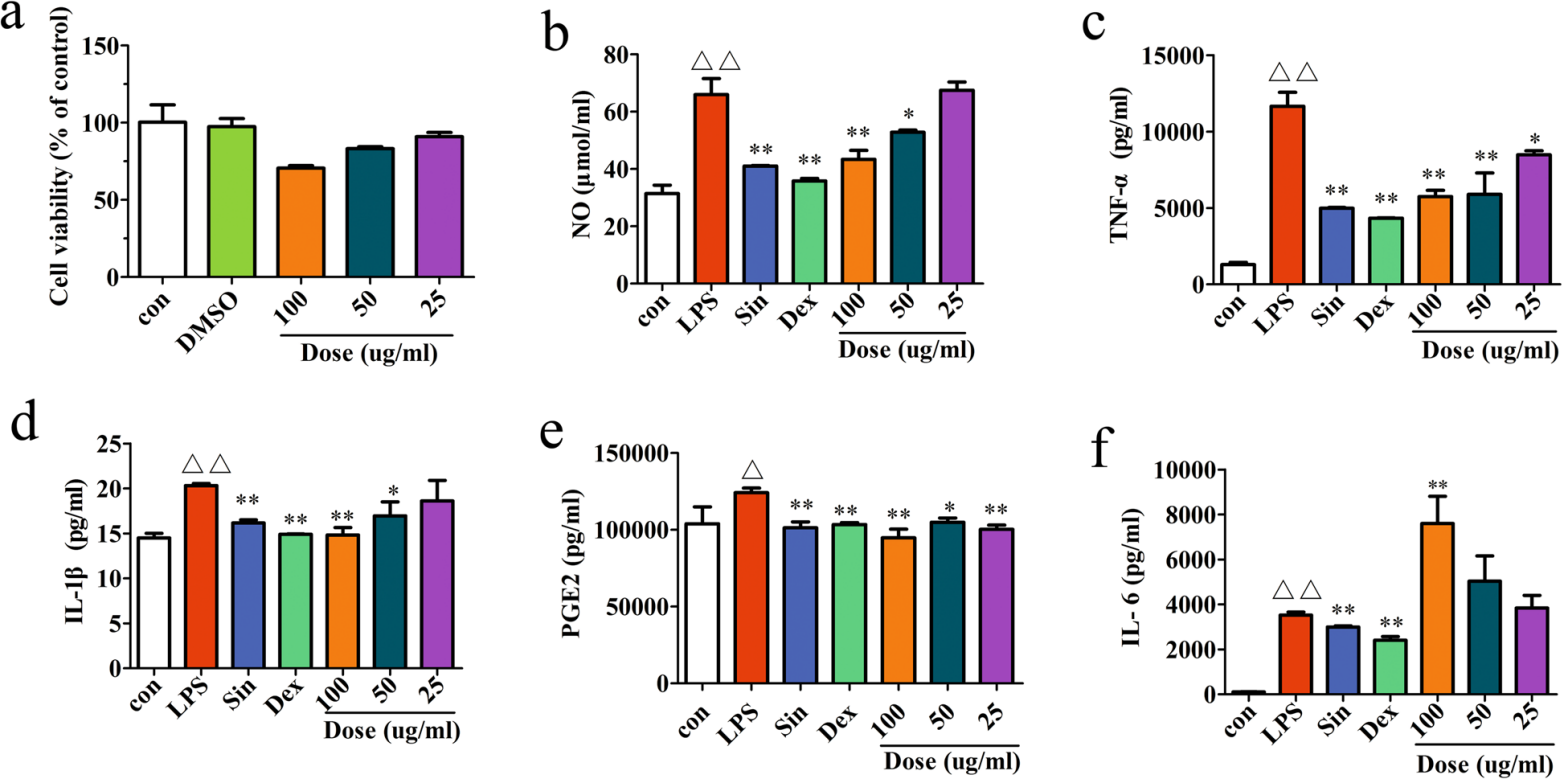
Effect of sino on the levels of inflammatory cytokines. **a** Cells viability at different concentrations of sino (25, 50, 100 μg/ml) was determined. Then, for the assay for NO (**b**), TNF-α (**c**), IL-6 (**d**), PGE_2_ (**e**) and IL-1β (**f**) with ELISA kits was determined. The values are presented are the mean ± SD (*n* = 3 in each group) of three independent experiments, ^*^p < 0.05, ^**^p < 0.01 compared with LPS group; ^△^*p* < 0.05, ^△△^*p* < 0.01 compared with con group

The potential cytotoxicity of sino was evaluated by the CCK assay. The result shoued that treatment with sino (25, 50, and 100 μg/ml) for 24 h did not cause significant cell viability change vs. normal control group (Fig. 2a). Thus, the effects of sino (25, 50, and 100 μg/ml) on RAW264.7 cells were not attributable to cytotoxic effects.

### Effect of sino on levels of inflammatory cytokines

The cellular activity of sino is shown in Fig. 2a. In this study, we investigated the effects of sino on the production of inflammatory cytokines in LPS-stimulated RAW264.7 cells. The results showed that sino inhibited NO (Fig. 2b), TNF-α (Fig. 2c), IL-1β (Fig. 2d) and PGE_2_ (Fig. 2e) in LPS-stimulated RAW264.7 cells in a dose-dependent manner in contrast, the mid and high doses of sino significantly promoted the release of IL-6 (Fig. 2f).

### Effect of sino on inflammatory response pathways

**Fig. 3:**
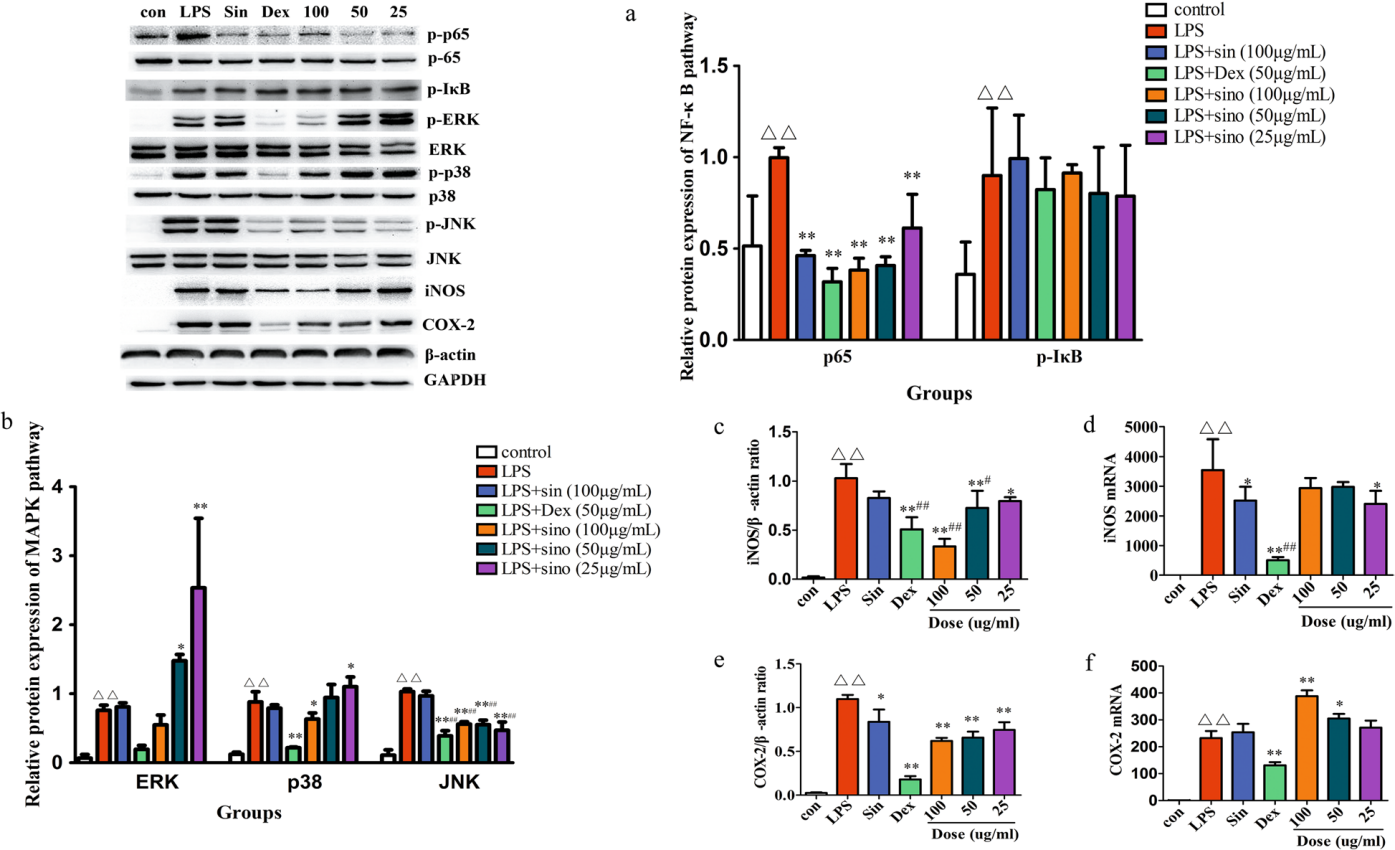
Effect of sino on inflammatory response pathways. Cells were pretreated with sin, Dex, and sino for 2 h, and then treated with 1 μg/ml LPS for 24 h. **a** Western blot analysis of phosphorylated NF-κB (**a**), phosphorylated MAPK (**b**), iNOS (**c**) and COX-2 (**e**) expression in RAW264.7 cells. qPCR analysis of iNOS (**d**) and COX-2 (**f**) in cells. The values are presented as the mean ± SD (n = 3), ^*^p < 0.05, ^**^p < 0.01compared with LPS group; △*p* < 0.05, △△*p* < 0.01 compared with con group, ;^#^*p* < 0.05, ^##^*p* < 0.01 compared with sin group

The MAPK and NF-κB signalling pathways have been reported to play a central role in the expression of proinflammatory cytokines such as TNF-α, IL-6, IL-1β and IL-1β in many cell types [26] . Another study also found that MAPK and NF-κB signalling pathways mediate the production of iNOS and COX-2 to affect the synthesis of NO and PGE2 [27]. Therefore, we investigated the effect of sino on the activation of NF-κB (p65 and IκB), MAPK (ERK, JNK and p38), iNOS and COX-2 by western blot, and detected the gene expression of iNOS and COX-2 by qPCR. Our results showed that sino significantly inhibited the phosphorylation of p65 in the NF-κB signalling pathway but had no significant effect on IκB phosphorylation (Fig. 3a). In the MAPK signalling pathway, sino (50, 25 μg/ml) promoted the phosphorylation levels of ERK and p38 (Fig. 2b), whereas it significantly inhibited JNK phosphorylation (Fig. 3b). Moreover, sino inhibited iNOS protein and gene expression levels (Fig. 3c, d), and COX-2 protein levels (Fig. 3e), while promoting COX-2 gene levels (Fig. 3f).

### Effect of sino on LPS-induced in acute lung injury

**Fig. 4:**
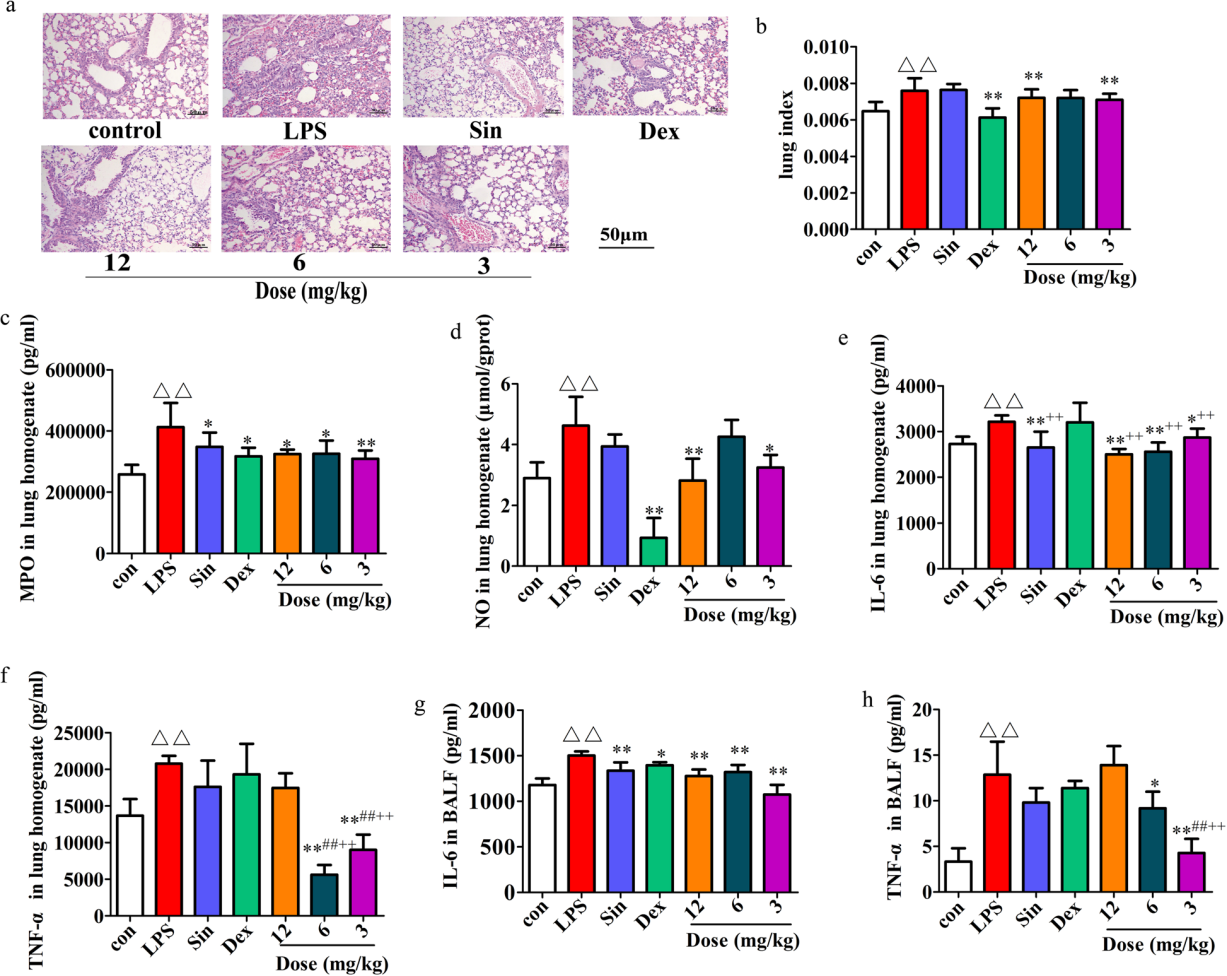
The protective effect of sino in ALI. After 12 h of LPS treatment in mice, sin, Dex, and sino were given for 12 h, pathological changes of lung tissue (**a**) were observed (200 ×), then LI (**b**) was calculated, moreover, the contents of MPO (**c**), NO (**d**), IL-6 (**e**) and TNF-α (**f**) in lung homogenate (*n* = 7 in each group) and the contents of IL-6 (**g**) and TNF-α (**h**) in BALF (*n* = 6 in each group) were determined according to the kits. Results are mean ± SD, ^*^p < 0.05, ^**^p < 0.01compared with LPS group; ^△^*p* < 0.05, ^△△^*p* < 0.01 compared with con group, ;^#^*p* < 0.05, ^##^*p* < 0.01 compared with sin group; ^+^*p* < 0.05, ^++^*p* < 0.01 compared with Dex group

Recent studies have found that inflammation plays an important role in ALI, and the occurrence of ALI can increase the release of proinflammatory cytokines, such as TNF-α and IL-6 [28]. Here, we examined histopathological changes that occur in ALI by H&E staining. The control group exhibited normal lung tissue structure in response to saline treatment, while the alveolar cavity of the LPS treated group was narrowed with punctured haemorrhage and inflammatory cell infiltration. However, sino treatment significantly attenuated these LPS-induced histopathological changes, suggesting that sino exerts a protective effect (Fig. 4a). For further confirmation, cytokines were assessed, and our results showed that when LPS was administered to mice for 24 h, the cytokine indices increased substantially, indicating that the model was successfully constructed. After treatment with sino, the LI (lung index) (Fig. 4b) and the release of MPO (Fig. 4c), NO (Fig. 4d), IL-6 (Fig. 4e) and TNF-α (Fig. 4f) in lung tissue were drastically inhibited. At the same time, the contents of IL-6 (Fig. 4g) and TNF-α (Fig. 4h) in BALF were also notably decreased.

## Discussion

Activation of the immune system leads to the occurrence of inflammatory reactions involving various mediators, such as NO, IL-1β, IL-6 and TNF-α [antipyretic, anti-inflammatory and ..]. Among them, IL-6 exerts both proinflammatory and anti-inflammatory effects in the inflammatory response. IL-6 is typically induced together with the proinflammatory cytokines TNF-α and IL-1β [29], but it is essential under normal physiological conditions and can inhibit the occurrence of various chronic liver diseases by selectively mediating signalling pathways [30]. IL-6 also plays a dual-functional role in the process of liver fibrosis-induced inflammation [31]. One study found that high levels of IL-6 in the circulation are a sign of poor prognosis in breast cancer, melanoma, and myeloma [32]. However, some studies have found that increasing the serum levels of IL-2, IL-6, IL-12, and TNF-α in tumour-bearing mice regulates the growth and differentiation of lymphocytes and activate macrophages, playing a regulatory role in antitumour immunity [33]. Meanwhile, Qinglong [34] studied the immunomodulatory effects of *Polyporus umbellatus* polysaccharide on macrophages and found that it promoted the secretion of inflammatory factors and induced significant immune enhancement. Their results revealed that sino promotes the production of IL-6, indicating that sino promotes immune enhancement, but the specific mechanism of action of sino in the immune pathway remains to be determined with respect to the interaction between immune signal molecules [34].

COX-2 expression in RAW264.7 cells at the protein level and gene level has been observed to be opposite to each other. The possible reasons for this phenomenon are as follows: (1) sino might act on protein translation rather than mRNA transcription in the process controlling COX-2 protein synthesis [35]. (2) sino may cause specific or non-specific degradation of protein, resulting in decreased protein content [36]. The specific reasons for these need to be further explored and analyzed by more experimental studies.

LPS binds to Toll-like receptor 4(TLR4) and transduces signals to activate multiple signalling pathways, including NF-κB and MAPK, ultimately leading to enhanced transcription of the proinflammatory cytokines TNF-α, IL-1β, and IL-6. IκB-α plays a key role in the NF-κB signal transduction pathway. When cells are stimulated by LPS, IκB kinase (IKK) is activated, IκB undergoes ubiquitination and exposes the binding site of IκB and NF-κB,at which point NF-κB is freed, the activity is enhanced, the expression of relevant inflammatory cytokines, inflammatory chemokines and inflammatory-related mediators is induced, and the range of inflammatory reactions is expanded [37, 38]. The NF-κB activation pathway described above, which relies on IKK to degrade IκB-α is referred to as the classical NF-κB activation pathway. Other unknown pathways such as the phosphoinositide-3-kinase (PI3K)/Akt pathway represent auxiliary activation pathways of NF-κB, which can bypass the phosphorylation of IκB-α and induce the dissociation of IκB-α from the trimer to activate part of NF-κB [39]. Our results revealed that sino did not inhibit IκB-α phosphorylation. We speculate that sino may achieve anti-inflammatory effect via other pathway rather than the classic IKK/IκB/NF-κB pathway, which is worth investigating. At the same time, studies have found that MAPK-specific inhibitors may alter the activity of other kinases, i.e., activation of different MAPK signalling pathways is dynamically regulated. When some of these signalling pathways are inhibited, other signalling pathways may be activated [40]. Therefore, it is possible that sino promotes the phosphorylation of ERK and p38 due to feedback regulation between different MAPK signalling pathways. Meanwhile, Lu [41] found that astilbin upregulates LPS-induced JNK phosphorylation, suggesting that it might be associated with M1 (classical activation) and M2 (alternative activation) polarization of RAW264.7 macrophages. However additional experiments are needed to elucidate the anti-inflammatory mechanism of sino.

At present, it is believed that the most essential pathogenesis of ALI is caused by uncontrolled inflammatory reactions in the lung and imbalances between the proinflammatory and anti-inflammatory systems. When inflammation occurs, neutrophils and monocytes infiltrate into inflammatory tissues in large quantities. MPO is a functional marker and activation marker of neutrophils, and its levels and activity represent the function and activation state of neutrophils [9]. In this study, sino significantly reduced MPO activity, indicating that sino might inhibit inflammation in ALI by simultaneously attenuating the accumulation of neutrophils. TNF-α and IL-6 not only directly affect cellular production of damage but also attract inflammatory cells such as monocytes, macrophages and neutrophils to aggregate inflammatory mediators such as prostaglandins, lysosomal enzymes and hydrogen peroxide, inducing or aggravating ALI, pulmonary oedema and sepsis [42, 43]. The results of this study revealed that sino exerts a significant inhibitory effect on TNF-α and IL-6, indicating that sino might achieve an anti-inflammatory effect on ALI in mice by inhibiting TNF-α and IL-6. However, these results were completely opposite in vitro. This may be because sino regulates the inflammatory response through a variety of mechanisms in vivo, but this needs further research in future experiments.

It is worth noting that sino exhibited stronger anti-inflammatory activity than sin based on NO, TNF-α, iNOS and MAPK results, which might be caused by the position exchange of methoxy and carbonyl groups in the structures. However, there are also some deficiencies. For example, ALI has a variety of pathogenetic mechanisms. Whether sino protects the lung from LPS-induced injury through other mechanisms requires further exploration. Overall, sino has been demonstrated to be a leading compound worthy of further study.

## Conclusion

In summary, our demonstrated that sino effectively reduces the release of inflammatory factors in vitro and in vivo, potentially through the inhibition of the NF-kB and JNK signalling pathways. We also confirmed that sino attenuates the inflammatory response in an ALI mouse model induced by LPS, indicating that sino may be a potential therapeutic strategy for the treatment of other inflammatory response-related diseases.

## Supplementary Information

**Additional file 1.**

## Data Availability

The datasets used and analysed during the current study are available from the corresponding author on reasonable request.

## Abbreviations

Sino: Sinoacutine
LPS: Lipopolysaccharide
NO: Nitric oxide
TNF-α: Tumor necrosis factor-α
PGE2: Prostaglandin E2
iNOS: Nitric oxide synthase
COX-2: Cyclooxygenase-2
JNK: c-Jun NH2 terminal kinase
ERK: Extracellular signal regulated kinase
MPO: Myeloperoxidase
BALF: Bronchoalveolar lavage fluid
ALI: Cute lung injury
ARDS: Acute respiratory distress syndrome
SAIDs: Steroidal anti-inflammatory drugs
NSAIDs: Non-steroidal anti-inflammatory drugs
Dex: Dexamethasone sodium phosphate injection
Sin: Zhengqingfengtongning injection
LI: Lung index
