# Supplementary material for: Sinoacutine inhibits inflammatory responses to attenuates acute lung injury by regulating NF-κB and JNK signaling pathways
Source: BMC Complement Med Ther. 2021 Nov 20;21:284. doi: 10.1186/s12906-021-03458-0 (PMC8605577; doi:10.1186/s12906-021-03458-0)

Sinoacutine Inhibits Inflammatory Responses to Attenuates Acute Lung Injury by Regulating NF-κB and JNK Signaling Pathways

Yuancui Zhao[1, 3+], Lili Cui [4+] ,XingXin Yang[5], Xingqian Sun[1, 2], Yunkuan Liu[1, 3] Zixian Yang[1], Liyuan Zhu[1], Chaorui Peng[1], Danye Li[1], Junfei Cai[1] ,Yunshu Ma[1,2*]

COX-2

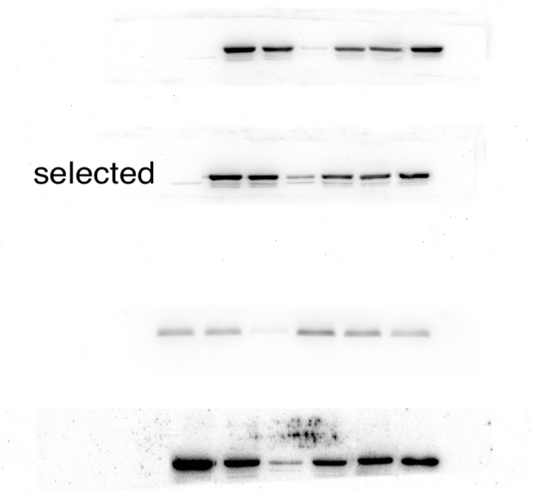

ERK

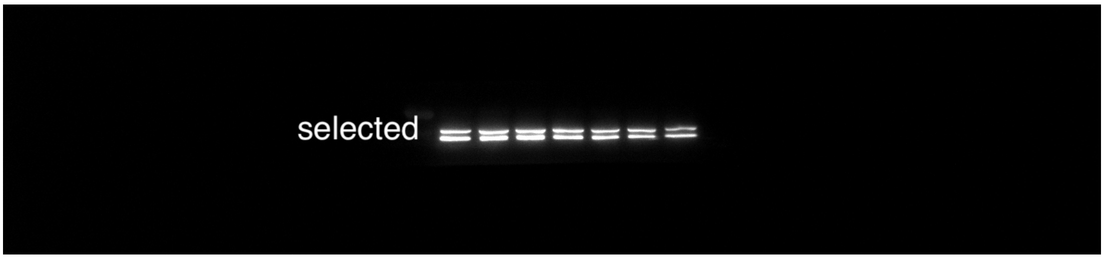

GAPDH

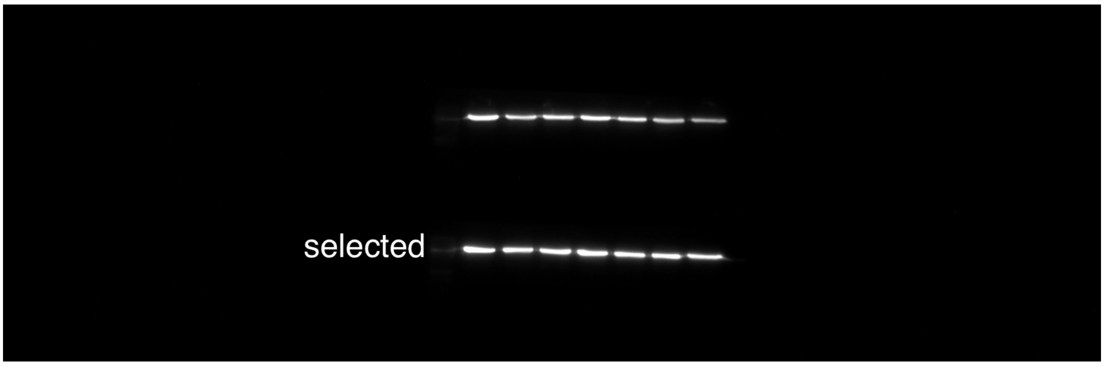

iNOS

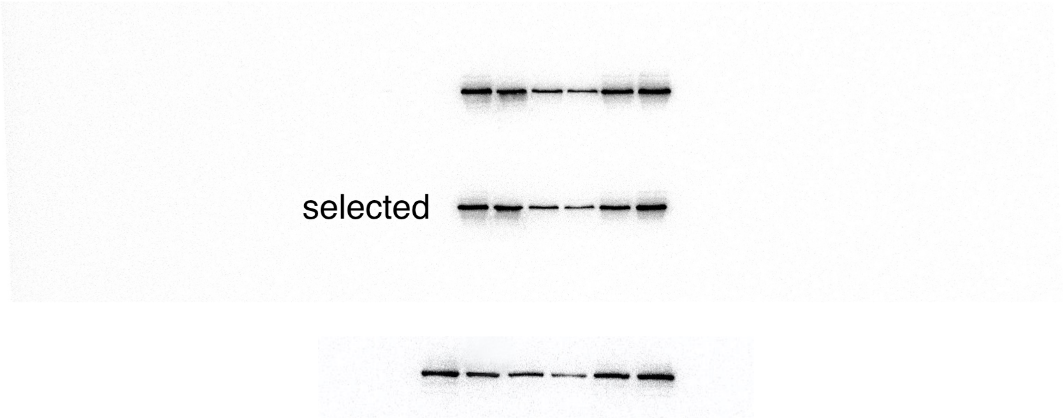

JNK

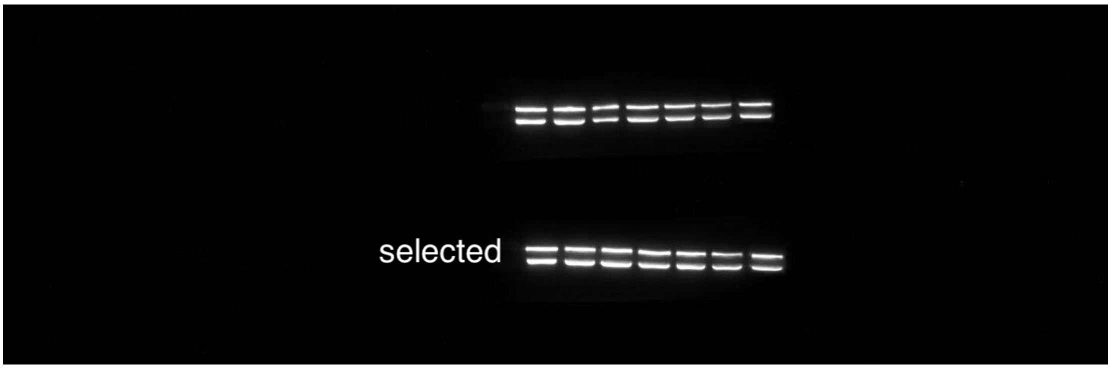

p-ERK

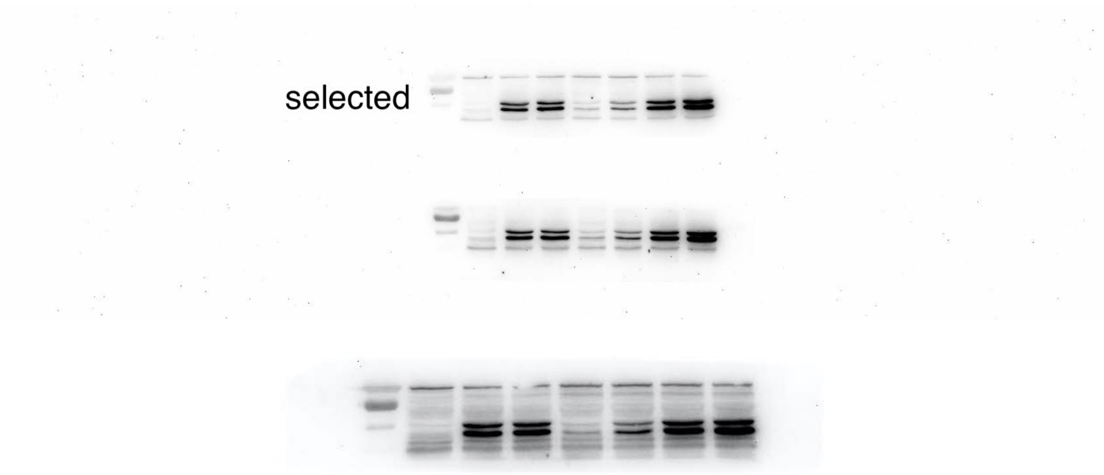

p-IκB

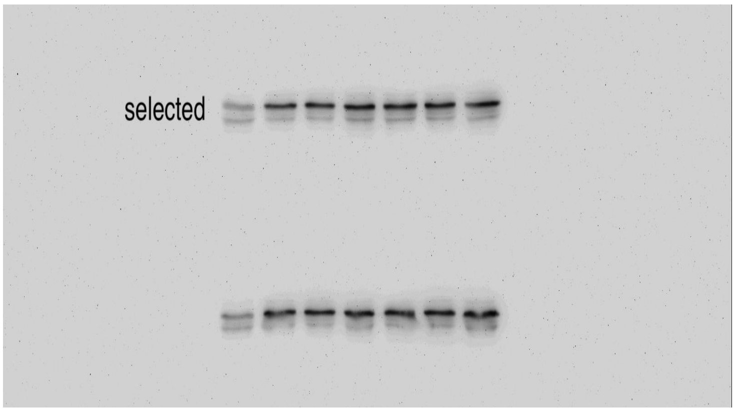

p-JNK

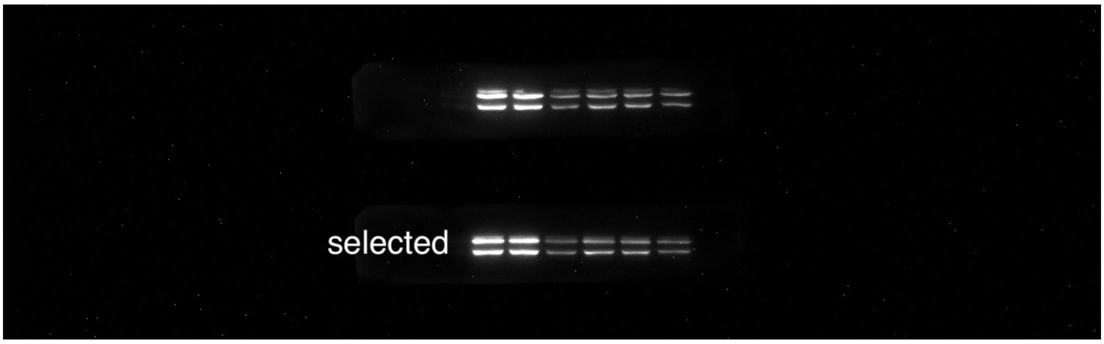

p-p38

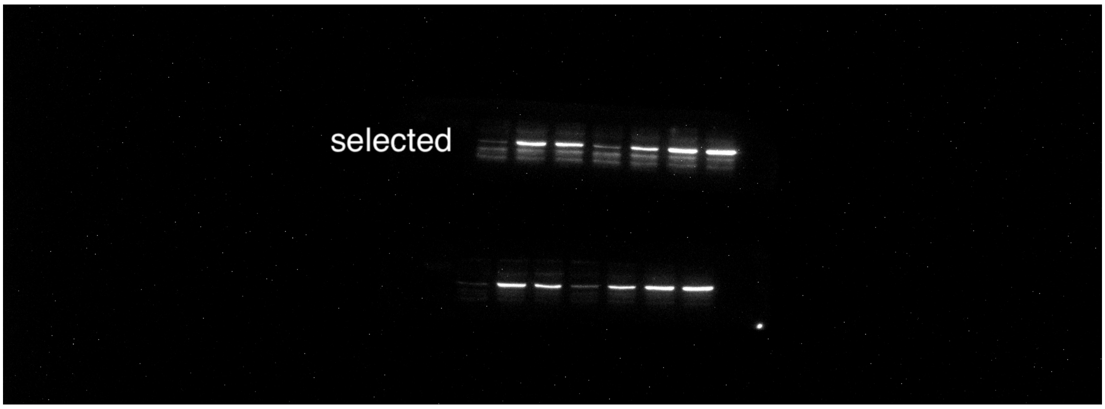

p-p65

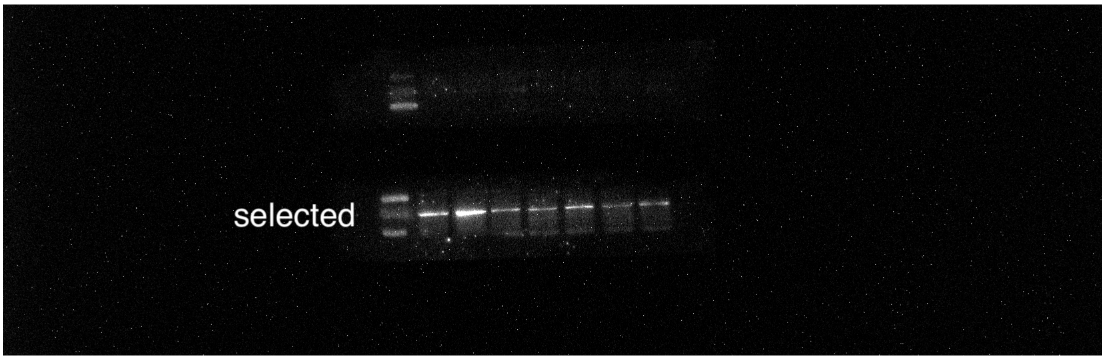

p38

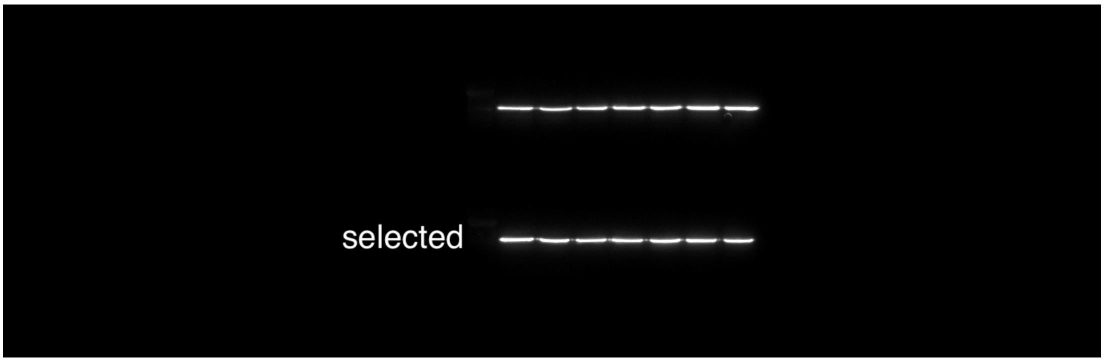

p65

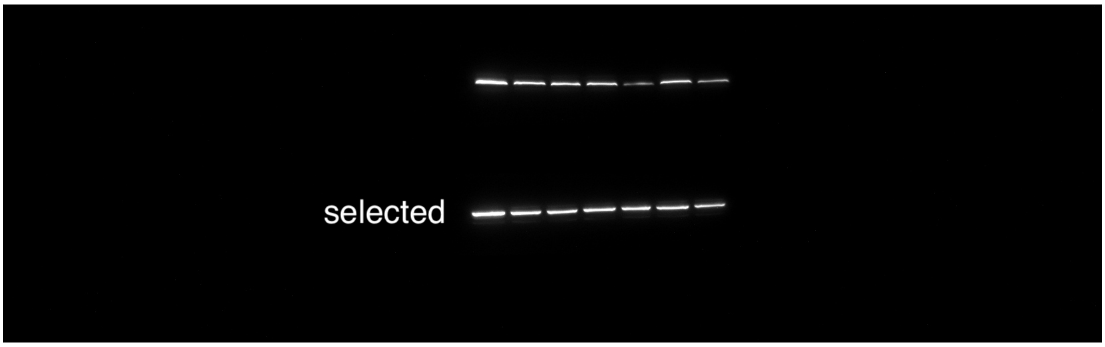

β-actin

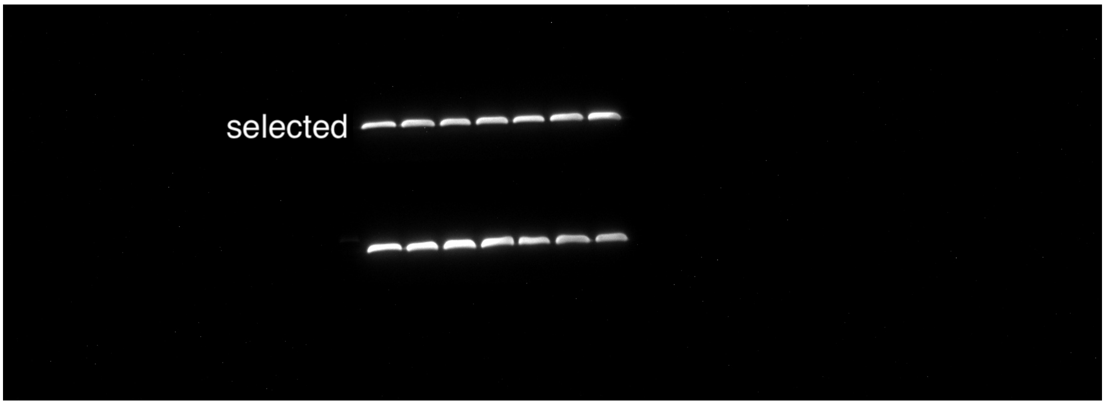

Supplement: Supplementary file 1 — Additional file 1. [file 12906_2021_3458_MOESM1_ESM.pdf]
